# Applicability of general grief theory to Swedish women's experience after early miscarriage, with factor analysis of Bonanno's taxonomy, using the Perinatal Grief Scale

**DOI:** 10.3109/03009731003739851

**Published:** 2010-07-19

**Authors:** Annsofie Adolfsson, Per-Göran Larsson

**Affiliations:** ^1^School of Life Sciences, University of Skövde, SkövdeSweden; ^2^Department of Obstetrics and Gynecology, Skaraborgs sjukhus, Kärnsjukhuset Skövde, SkövdeSweden; ^3^Division of Women and Child Health, Department of Clinical and Experimental Medicine, Faculty of Health and Sciences, Linköping University, LinköpingSweden

**Keywords:** Content analysis, factor analysis, general grief theory, miscarriage, perinatal grief scale, women

## Abstract

**Background:**

Grief is a normal phenomenon but showing great variation depending on cultural and personal features. Bonanno and Kaltman have nonetheless proposed five aspects of normal grief. The aim of this study was to investigate if women with miscarriage experience normal grief.

**Material and methods:**

Content analyses of 25 transcribed conversations with women 4 weeks after their early miscarriages were classified depending on the meaning-bearing units according to Bonanno and Kaltman's categories. In the factor analyses, these categories were compared with the Perinatal Grief Scale and women's age, number of children and number of miscarriages, and gestational weeks.

**Results:**

Women with miscarriage fulfill the criteria for having normal grief according to Bonanno and Kaltman. All of the 25 women had meaning-bearing units that were classified as cognitive disorganization, dysphoria, and health deficits, whereas disrupted social and occupational functioning and positive aspects of bereavement were represented in 22 of 25 women. From the factor analysis, there are no differences in the expression of the intensity of the grief, irrespective of whether or not the women were primiparous, younger, or had suffered a first miscarriage.

**Conclusion:**

Women's experience of grief after miscarriage is similar to general grief after death. After her loss, the woman must have the possibility of expressing and working through her grief before she can finish her pregnancy emotionally. The care-giver must facilitate this process and accept that the intensity of the grief is not dependent on the woman's age, or her number of earlier miscarriages.

## Introduction

Grief is a common and unavoidable trauma. Its intensity and duration is unique to the individual. Some people grieve intensively for a short time, some grieve moderately for longer periods, and still others are quick to resolve their grief. This variation makes it difficult to define normal, excessive, or inadequate grief, or to say what constitutes complicated grief. Grief as a topic has been studied and explained within different scientific disciplines by different authors. Some of those frequently quoted are Raphael and Wilson (1), Parkes (2), Stroebe et al. (3), and Worden (4). Bonanno and Kaltman (5) have provided a synthesis of all these authors and presented a comprehensive theory.

Bonanno and Kaltman (5) describe grief as a normal reaction to lasting stress. Grieving is a normal phenomenon but with a variety of cultural and emotional features. Bonanno and Kaltman have nonetheless proposed five aspects of grief and the grieving process. Normal grief exhibited by 50%–85% of those grieving involves moderate disruption in cognitive, emotional, physical, or interpersonal functioning during the first month after the loss. On the other hand, a small minority (15%) experience only what may be described as minimal grief. After a year most people (85%) have resumed their base level of functioning, while 15% show some form of chronic grief and symptoms such as depression, anxiety, or post-traumatic stress disorder (PTSD).

Expressing grief is necessary, and those grieving must work through their memories, their thoughts and feelings, at the same time venting the pain of their loss and releasing themselves from attachment to the deceased (5). The word grief is often substituted by an alternative term such as mourning or bereavement (6).

Bonanno and Kaltman's study describes five aspects of the grieving process:

The first aspect is cognitive disorganization, which is when a grieving person initially finds it difficult to accept the reality that they have lost something. This is combined with a sense of abandonment, disorder, and preoccupation with their loss. Twenty percent of grieving people have difficulty making decisions and maintaining concentration, or they tend to make more mistakes than usual in the first initial month after the loss. Grieving persons have worries and concerns associated with their identities. In the first month they feel uncertain regarding their prospects for the future. It is common for a quest for meaning to begin in order to resolve the explanation for the loss. They may describe the world as being less meaningful than before their loss.

The second aspect is dysphoria, which is emotional stress. It is often manifested in signs of remorse, irritability, sorrow, guilt, fear, and hostility, followed by sadness and resignation. Less often there is anxiety, shame, and guilt, along with repugnance, envy, fear, and jealousy. Distinct yearning or pining for the dead person may occur. Feeling lonely even in the company of others is common. There are two forms of loneliness, social and emotional. Social loneliness is described as a general feeling of solitariness, absence of commitment to a social network, and a sense of marginalization. Emotional loneliness is an absolute perception of missing someone to divulge one's feelings to and to share those feelings with. The latter causes a profound form of inner isolation and a feeling of discontent and frustration.

The third aspect is that of health deficits. Grief may be accompanied by physical symptoms in any combination of the following, such as shortness of breath, cardiac palpitations, difficulty in digesting food, with loss of appetite and restlessness and insomnia. Grief can also be linked to an increase in mortality during the first year after a severe loss. There is evidence that a relationship exists between bereavement and a brief compromise in immune functioning.

The fourth aspect of the grieving process is disrupted social and occupational functioning. Social activity decreases for roughly half of grievers during the first month of a normal grieving process. Bereaved people experience difficulties in their occupational roles and dissatisfaction with their work performance. Their expression of the pain of grieving evokes self-defense reactions in others. Role identification may be difficult, and this applies particularly to parents or custodians of the deceased.

The fifth and final aspect is the positive feature of the grieving process. Grief can motivate changes in personal identity. As the grieving process takes its path the grieving person is exposed to much that is new: a new identity, new perception of life, invigorated passion for life, and humility. Some people also realize a new sense of freedom after their loss. Positive thoughts about the future can be experienced. Such feelings and thoughts are key aspects in the grieving process. Smiles, laughter, and a positive mental attitude help to accelerate the process (5).

Bonanno extends the general theory of grief to other losses as well. Examples are the loss of employment or losing a child to mental illness or intellectual disability. Grief is a general and broad emotion which should be explored from the perspective of different types of losses and from the perspective of different cultures. He claims that through using his taxonomy it is possible to test and investigate whether grief is general and whether it emerges in response to other types of loss and in terms of other cultures as well (5).

### Miscarriage and loss

Early miscarriage is the loss of a fetus before 13 weeks of gestation, irrespective of cause. In a complete miscarriage the products of conception are completely expelled. On the other hand, in incomplete miscarriage all of the products of conception are not completely expelled. In a missed abortion the pregnancy termination takes place but nothing is expelled. The cause of most miscarriages is fetal death due to fetal genetic abnormalities that are usually unrelated to the mother. Other possible causes of miscarriage include infection, physical disabilities, hormonal factors, immune response, and serious systemic diseases such as diabetes or thyroid disorders (7). Signs of miscarriage are pain in the lower back, pain in the abdomen and vaginal bleeding with or without abdominal cramps. Diagnosis is performed after a pelvic examination followed by transvaginal ultrasound. The treatment varies depending upon the nature of the miscarriage. For a complete miscarriage no further measures are required. An incomplete miscarriage is treated with expectant management for 5 to 7 days, and if all of the products of conception are not expelled a dilation and curettage (D&C) is performed (8,9). Missed abortion is treated with curettage or misoprostol (10).

A woman who suffers a miscarriage loses not only the embryo or the fetus but she also loses the dreams for a future and the plans for her eventual child (11). The experience of miscarriage may also be traumatic for her, because she incurs physical pain and bleeding as well and may be required to undergo a further procedure, as in a D&C (12). A woman's experience of miscarriage is described as grief with grieving symptoms (13,14). Symptoms of depression in post-miscarriage are found in between 45%–50% of the women during the first few weeks after the loss (15,16). Anxiety has been measured and identified after miscarriage (17,18), and even post-traumatic stress has been identified (19).

The primary aim was to investigate whether women after miscarriage experience grief reactions similar to those that accompany general grief. The secondary aim was to test in a factor analysis as to whether the Bonanno categories are correlated in any way with maternal age, number of children and number of miscarriages, week of pregnancy, nature of miscarriage, and whether there is any correlation with the Perinatal Grief Scale (PGS).

## Material and methods

During the study period August 2002 to May 2003, 25 follow-up visits of a total of 147 follow-up visits by women who had early miscarriages, which is defined as a miscarriage occurring before 13 weeks gestation, were documented for this study at the department of Obstetrics and Gynecology in the Central Hospital in Skövde, Sweden.

All of the women who experienced miscarriage were invited to the follow-up visit with a midwife and randomized into one of two groups. One group was a structured consultation based on Swanson's middle-range caring theory, which says that the midwife should be knowing, doing, being, maintaining belief, and enabling the women who have suffered a miscarriage (23). The other group was the control group, which participated in a simple consultation (20). The 25 women that were documented for the study partook of the structured consultation based on Swanson's theory. The women signed a written consent form before the visit. The follow-up visits took place 4 weeks after the woman had been diagnosed of having a miscarriage. At the visit she was given a semi-structured interview about her experience of miscarriage and her resulting emotions. They were also evaluated in terms of active grief, difficulty coping, and despair by means of a PGS questionnaire. The PGS is a grief scale adapted and recommended for use after miscarriage (21,22). This includes encouraging the patient's self-esteem and having a positive mental attitude tempered by a midwife's realism based upon her knowledge and experience (23,24). If the woman agreed, the consultation was tape-recorded for later analysis and performed anonymously. The recording had no influence on the consultation. The 25 interviews which were recorded on tape lasted between 20 minutes and 100 minutes and averaged 45 minutes.

### Data analysis

The tape-recorded conversations were transcribed verbatim. The text was then analyzed in several stages by the first author (A.A.), and the coding was discussed and performed together with the second author (P.-G.L.). First each interview was read as a whole. Meaning-bearing linguistic units were picked out, and those with similar meaning were grouped together (25). These units ranged from a few words to several lines of text (26). Each meaning-bearing unit was given a code based on aspects of Bonanno and Kaltman's general grief theory. All units were divided into five categories corresponding to the five aspects of their general grief theory: cognitive disorganization, dysphoria, health deficits, disrupted social and occupational functioning, and positive experience of bereavement (5); see Table I. The meaning-bearing units in the five categories were summarized. The frequency of meaning-bearing units was assumed to be a measure of their respective categories' importance to the individual woman concerned and also related to how much grief the woman was experiencing.

**Table I.** The five aspects of Bonanno's general grief theory: cognitive disorganization, dysphoria, health deficits, disrupted social and occupational functioning, positive aspects of bereavement, with coding of meaning-bearing units as used in the content analysis.

Common time-limited disruptions in functioning in the grief process	
1. Cognitive disorganization	Decision-making difficulties Concentration difficulties Making more mistakes than usual Identification disturbance ‘A piece of me is missing’ Sense of disrupted future ‘The world is less meaningful’ ‘Why me?’ Search for meaning
2. Dysphoria	Emotional malaise Dysphoric emotion Distress, anger, irritability, hostility, sadness, fear, guilt Grief, anger, guilt, fear Lower frequency: anxiety, shame, guilt, disgust, envy, fear, jealousy Pining and yearning Social loneliness Emotional loneliness
3. Health deficits	Increased visits to doctor Shortness of breath Palpitations Digestive problems Loss of appetite Restlessness Insomnia Pain in lower abdomen and bleeding
4. Disrupted social and occupational functioning	Withdrawal from social activity Negative impact from others Role disruptions Difficulties with new relationships
5. Positive aspects of bereavement	Positive change in identity New identity New opinion Emphasis on future goals Showing a humble spirit New freedom Positive emotion Good care

### Statistical analysis

Factor-reduction analysis was performed using the SPSS program 12.0 (SPSS Inc., Chicago IL) for the following purposes: identifying underlying, concealed, or latent factors of possible explanatory value and finding correlation among a set of variables and testing hypotheses concerning variables and structures (27).

Correlations among variables were investigated, and values above 0.5 were deemed to indicate associations. The variables tested were age, number of children, number of miscarriages, week of pregnancy, nature of miscarriage (missed abortion or other miscarriage diagnosis), PGS scores for active grief, difficulty coping, and despair. They were correlated with the number of meaning-bearing units from the Bonanno respective taxonomic categories (cognitive disorganization, dysphoria, health deficits, disrupted social and occupational functioning, and positive experience of bereavement). Factor totals above 0.5 were deemed to be statistically significant.

### Ethical considerations

The study was approved by the regional ethics committee of the University of Gothenburg.

## Results

Mean gestational age at the time of the miscarriage was 9 weeks (range: 5–13), and the mean age of the women was 31 years (range: 20–40). A total of 8 women were primiparous; 12 had given birth previously; and 6 of the 25 women had prior experience of miscarriage.

### Content analysis

For all of the 25 women, three of the Bonanno categories of grief are represented in the content analysis (Table II). These are cognitive disorganization, dysphoria, and health deficits. Disrupted social and occupational functioning and positive experience of bereavement were each represented in 22 of 25 women (Table II).

**Table II.** Frequency of coded meaning-bearing units for each woman interviewed, based on Bonanno's taxonomy and the total of bearings units.

ID and taxonomic cross-tabulation count						
	Taxonomic categories					
Woman's ID	Cognitive disorganization	Dysphoria	Health deficits	Disrupted social functioning	Positive experience	Total
1	9	16	3	8	9	45
4	30	31	3	4	5	73
10	33	24	12	8	3	80
12	13	35	2	5	7	62
17	45	49	26	11	2	133
24	17	11	6	10	4	48
27	35	33	2	7	6	83
31	6	6	4	3	1	20
36	16	12	6	8	0	42
48	37	24	6	17	1	85
63	24	30	4	10	12	83
71	17	7	14	4	4	46
73	24	31	5	12	4	76
79	14	22	11	18	6	71
80	11	10	2	3	3	29
82	29	24	7	3	22	85
91	5	14	4	5	0	28
98	18	25	4	2	5	54
101	31	63	2	13	1	110
105	19	19	6	5	0	52
106	26	35	6	4	2	73
112	21	32	10	6	6	83
119	10	6	12	0	7	35
122	3	2	4	0	5	14
130	7	10	7	0	4	28
Totals	500	571	168	166	119	1524

Cognitive disorganization was found 20.0 times per woman on average. For example, all the women's energies and thoughts were focused on the prospective child, and they feared that they would never get pregnant again. They felt they had missed their chance of becoming a mother before becoming too old.

I was looking forward … it was our first child, would have been our first child, and we were looking forward to it so incredibly, saw the future very much as a great thing, to us it was the pregnancy, now we only have each other (interview #12/lines 134–136) including all subsequent quotes ).

Many women described their sense of abandonment and loneliness in relation to the care services. They often blamed themselves for something they had done or eaten.

The code for dysphoria was registered 22.8 times on average. Women described feelings of being abandoned or marginalized and that they experienced loneliness during the consultation.

NN said to me that ‘nothing was produced this time, better luck next time …’ (63/530-531).

Later the women experienced internal stress and fatigue and found it difficult to talk about their experience. They felt lonely.

Health deficits were coded on average 6.7 times, examples being bleeding, lower abdomen or back pain, dizziness, insomnia, or restlessness.

I haven't had time to sit down and search my heart about this; I probably haven't wished to do so before now either (17/757-758).

Disrupted social and occupational functioning was coded 6.6 times on average. Examples are that the women experienced fatigue and emotional instability for longer periods than the members of their respective families regarded as normal. Remarks such as ‘Why is she so down? It's a whole week since the miscarriage’ were made. Reminders of child-birth and pregnancy were omnipresent in their immediate surroundings, in the community, and in the media. The women's ability to concentrate at work had deteriorated.

It is not the child … not the miscarriage … but everything together (91/218-219).

Positive experience of bereavement was registered on average 4.7 times per woman. This was confirmation that the women whom the staff responded to with empathy had a more positive experience of their miscarriage.

The women cited other examples of positive experiences in the grieving process. When the husband stood by his wife as her protector and supporter it provided her with a sense of security. Conversations with other women demonstrated that they shared similar experiences of miscarriage, which validated their feelings.

We could be pregnant together (119/308-309).

I became aware of different sides of my partner because he was so careful and caring when I had pain and bleeding (79/339-346).

### Factor-reduction analysis

**Figure 1.**
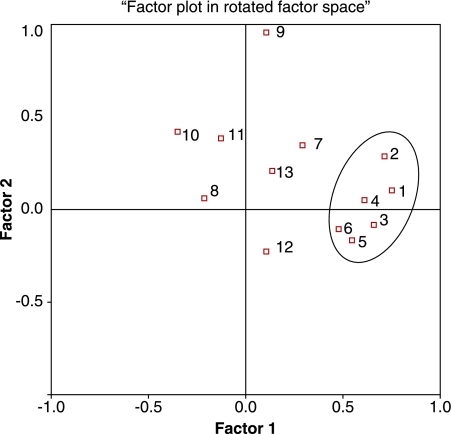
Factor analysis with two factors and orthogonal (varimax) rotation of the 13 variables, 5 from Bonanno taxonomy, 3 from the sub-scale of Perinatal Grief Scale (PGS), and 4 background variables, as well as sub-diagnosis of miscarriage. The variables contained in the circle have statistical significance and vary in correlation. The PGS: despair, difficulty coping, and grief; as well as Bonanno: dysphoria, cognitive disorganization, and disrupted social functioning are of clinical importance and varied in correlation statistically. (1 = PGS despair; 2 = Dysphoria; 3 = PGS difficulty coping; 4 = Cognitive disorganization; 5 = Disrupted social functioning; 6 = PGS grief; 7 = Number of miscarriages; 8 = Positive aspects; 9 = Age; 10 = Diagnosis; 11 = Gestational week; 12 = Health deficits; 13 = Number of children).

Factor-reduction analysis gives rise to two variables. It demonstrated that there is no statistical correlation between the amount of grief a woman feels and her age or whether or not she already had children. The following did not have any influence in the factor analysis either: prior experience of miscarriage, nature of miscarriage, or week of pregnancy. Factor-reduction analysis did show a correlation between certain components of grief described in Bonanno and Kaltman's terms and by means of PGS sub-scales. In the correlation matrix, dysphoria and the PGS despair sub-scale are correlated (*r* = 0.510). The loads per factor of the respective variables are shown in Table III. The factor loading for each of both the factors is 0.995, and the two factors with eigenvalue above 2.0 explain 40% of total loading. The statistically significant variables are cognitive disorganization, dysphoria, disrupted social functioning, and the PGS difficulty coping and despair sub-scales. Bonanno and Kaltman's taxonomy and the PGS sub-scales were Factor 1, while the women's age, diagnosis, and week of pregnancy were Factor 2. On the other hand, the number of miscarriages was bifactorial, meaning that it affected both factors. The factors were plotted in rotated factor space (Figure 1). The PGS despair, difficulty coping, grief, the Bonanno aspects of dysphoria, cognitive disorganization, and disrupted social functioning were of clinical importance and varied in correlation statistically.

**Table III.** Variable load per factor. Extraction method: principal axis factoring. Attempt to extract two factors, requiring more than 25 iterations. (Convergence = 1.046E−03.) Extraction was terminated. Factors with eigenvalues greater than 2.0 explain 40% in a two-factor structure. The factor transformation matrix after varimax rotation is the same for factors 1 and 2, i.e. 0.995. PGS - Perinatal Grief Scale (22).

	Factor	
Factor matrix	1	2
PGS despair	0.757	
Dysphoria	0.738	0.215
PGS difficulty coping	0.648	0.149
Cognitive disorganization	0.614	
Disrupted social functioning	0.524	−0.219
PGS grief	0.462	−0.154
Number of miscarriages	0.324	0.317
Positive aspects	0.205	
Age	0.201	0.940
Diagnosis	0.306	0.455
Gestational week		0.394
Health deficits		−0.238
Number of children	0.157	

## Discussion

By classifying grief according to the Bonanno taxonomy and measuring it according to the number of meaning-bearing units, one can obtain a measure of grief and its intensity and a measurement of the difficulties encountered in the experience of grief. Then this grief is comparable among different groups and cultures. In Sweden, women who experience early miscarriage have been found to undergo a grief process in accordance to the general principles of grief as formulated by Bonanno and Kaltman (5). Our material bears out our hypothesis, and that is that women whose miscarriages took place early in pregnancy were found to undergo normal grief (primary outcome variable). Most meaning-bearing units from the tape-recorded conversations fall into the categories of cognitive disorganization and dysphoria (Table II).

What distinguishes grief in women who have suffered miscarriages is that there are no memories, there is no object to grieve, and other people are unaware of the woman's loss. This was in contrast to the loss of a close relative or living person (28,29). The grief process after miscarriage is more rapid than after other losses. Following early miscarriage the most intensive grief lasts for a few days, perhaps up to a week, and most of the grief process is over after 4 to 6 weeks. After 4 months most women have emerged from the normal grief process (28,29). In a previous Swedish study (11), the women reported that they stayed in bed at home for a few days because they had abdominal or back pain and were tired. After that life had to go on. A third of the women (21) were absent from work for only 1 day because of the miscarriage. With our knowledge of the grief process the woman should consider taking a few days' leave in order to be able to react to and work through their loss in their own way before resuming their normal lives. In this way resurgence of reactions to the loss in other subsequent situations may be avoided. Staying home from work for only a day is not enough time to work through the loss associated with a miscarriage. In Sweden it is not acceptable to be emotionally down for more than a week according to the family culture. A miscarriage should be accepted and resolved in order for a couple to attempt a new pregnancy without unresolved grief reactions from the miscarriage. Many couples are ready to attempt a new pregnancy 4 to 5 weeks after the miscarriage, when the woman has her first menstrual period (30).

As a secondary outcome, variable factor analysis was performed showing that the number of children, the woman's age, the week of pregnancy, and the number of miscarriages are unrelated to the intensity of the grief as expressed in the number of meaning-bearing units. Women who had never miscarried before experienced and described as much grief as those women who had suffered more than one prior miscarriage. The younger women showed as much grief as the older women. The factor analysis did not reveal any correlation or relationship to the number of children the women had and their intensity of grief feelings. This is in agreement with the findings of other studies (31).

In the factor analysis, Bonanno and Kaltman's difficult and complicated taxonomy combined with the considerably easier and quicker PGS and its sub-scales together make up one factor (Table III, Figure 1). PGS measures the grief of the woman in relation to her social network and functioning. The Bonanno and Kaltman taxonomy measures the manifestations of the grief in the woman. Nonetheless, there is congruence between the Bonanno and Kaltman categories and the PGS, with dysphoria in the Bonanno taxonomy and the despair PGS sub-scale being particularly closely correlated (Table III). PGS as adapted to perinatal losses is simpler to answer and to analyze (21,22).

Early miscarriage may be the greatest experience of stress the woman has experienced in her life to date and may be the first time she has suffered a painful loss (32). Many women blame themselves for their miscarriages (11). If the pregnancy was postponed and planned to take place only after the education of the woman, for example, her self-reproach increases. Sometimes she directs her reproaches against the man. She may misinterpret his attempts to be supportive. She may focus her anger on the man who in turn feels powerless (4). The sense of being alone in her grief could be mitigated if others accepted the fact that she is grieving her loss and that the loss represents more than just an embryo or fetus from the perspective of the woman. The empty amniotic sac represents the termination of a dream for an expected child in the woman and also emphasizes that a state of being has perished with the miscarriage.

In this study there was no correlation between missed abortion and the intensity of grief in terms of the number of meaning-bearing units in the content analysis which had previously been identified (20). The previous study showed that women who had suffered a missed abortion had even higher PGS scores 4 months after early miscarriage than they did after 4 weeks. That survey was carried out 4 months after the miscarriage, and this study is based only on the structured consultation that took place 4 weeks after the miscarriage.

Many of the women described their sense of abandonment and loneliness in relation to care services (cognitive disorganization). The women called their local maternity center, emergency department, and gynecological clinic repeatedly to set up an appointment for an investigation of their bleeding and subtle feeling that something was wrong with the pregnancy. They were told to wait and see and to come back if their bleeding or abdominal pain became more intense. These symptoms appear immediately after a complete or incomplete miscarriage but in the case of missed abortion they are often delayed a few weeks. Sometimes the women found themselves having to wait a long time on the telephone in a queue or were asked to return to the clinic the next day. For women who suffered missed abortion the diagnosis took longer, and they believed that they had been pregnant for several weeks when their pregnancy was no longer viable. Improvement can be made in the health care sector with more attentive listening and being more proactive in asking about subtle symptoms and physical sensations.

In the semi-structured interviews the women described their feelings of being abandoned or marginalized by the system, and they described their feelings of experiencing loneliness during the consultation (dysphoria). The encounter with the gynecologist and the staff is based on a procedure and is lacking of empathy for the loss that the woman has experienced, which is so much more than the tiny embryo or fetus. This is also reflected in the perception of women with regard to the health care system (32). Other women have described gaps in the information provided by the health care system and the lack of empathy for those who have experienced miscarriage (18,28). The typical reaction among health professionals is to think of a miscarriage occurring in the first trimester as a rather common incident. Fully 20% of child-bearing women will experience a miscarriage before delivering their third child (33).

The organization of the health care system responsible for the caring of women experiencing miscarriage must first of all begin with the initial contact with the system when the woman is complaining of discomfort or bleeding, which are the first signs that something is not normal in her pregnancy. At this point in the system, it is very important that the staff listen attentively to the patient and are prepared to take the appropriate action, which probably would be an appointment with a doctor in due course. If the patient's concern is verified by the doctor with the diagnosis of miscarriage, this information must be communicated to the patient with a sense of compassion and responsibility for the patient's feelings and possible reactions to the diagnosis. As Worden (4) expresses it, miscarriage is the loss of a potential person, and this is why it is important for the grieving process to commence in a healthy manner. This process most importantly includes talking about the loss within the immediate social network of the patient. If this is insufficient for a particular patient's recovery, professional counseling should be provided. The content analysis provided confirmation that the women to whom the staff responded with empathy had a more positive experience of the miscarriage.

## Conclusion

The conclusion of our study is that the experience of a woman suffering grief after miscarriage is similar to the general grief that one may experience after the death of a close relative. General grief involves moderate disruption in cognitive disorganization, dysphoria, and social disruption. As women express normal grief it would be an error to use scales that measure depression, anxiety, or PTSD. Because of the overlap between grief and depression, the women will score highly on depression because of normal grief. The women must have the opportunity after their loss to express and work through their grief before they can resolve their pregnancies emotionally. The health care system must facilitate this process and accept that the intensity of the grief is not dependent on the age of the woman or the number of earlier miscarriages she may have experienced.
